# Repurposing a Detrimental Antibody Epitope as Targeted Therapeutics for Sepsis and Rheumatoid Arthritis

**DOI:** 10.21203/rs.3.rs-7633404/v1

**Published:** 2025-09-19

**Authors:** Weiqiang Chen, Li Lou, Xiaoling Qiang, Cassie Shu Zhu, Jianhua Li, Shujin Chen, Brian Xiong, Huan Yang, Ping Wang, Kevin J. Tracey, Haichao Wang

**Affiliations:** 1 The Feinstein Institutes for Medical Research, Northwell Health, 350 Community Drive, Manhasset, NY 11030, USA; 2 Departments of Emergency Medicine and/or Molecular Medicine, Donald and Barbara Zucker School of Medicine at Hofstra/Northwell, 500 Hofstra Blvd, Hempstead, NY 11549, USA

**Keywords:** HMGB1, Procathepsin L, Tetranectin, Sepsis, Arthritis, Peptide, Antibody, Epitope, Innate Immune Cells

## Abstract

Sepsis and rheumatoid arthritis (RA) are distinct yet mechanistically related conditions commonly driven by dysregulated inflammatory responses. Here, we explored the counterintuitive hypothesis that an epitope from a deleterious anti-tetranectin (TN) antibody (mAb9) could hold unforeseen therapeutic potential. By mapping mAb9’s epitope to P2 (residue 55–70), a region crucial for TN’s protective functions, we developed P2–1, a water-soluble derivative as a targeted therapy. P2–1 significantly improved survival and reduced systemic inflammation in a sepsis model, and attenuated arthritis severity and pain sensitivity in a RA model, even with therapeutic administration after disease onset. Mechanistically, P2–1 exhibited high-affinity binding to high mobility group box 1 (HMGB1) and selectively suppressed HMGB1-induced *Ctsl* mRNA upregulation and procathepsin L (pCTS-L) secretion from human immune cells, crucially without perturbing other HMGB1-induced cytokines and chemokines. We further validated pCTS-L as a therapeutic target by demonstrating that a neutralizing antibody conferred potent anti-arthritic effects, reducing joint inflammation, pain, and structural damage. Our findings introduce a paradigm-shifting drug discovery strategy that transforms insights from paradoxical antibody action into targeted therapeutics for the HMGB1-pCTS-L axis, not only delivering P2–1 as a potent therapy but also establishing pCTS-L as crucial mediator of inflammatory diseases like sepsis and RA.

## One Sentence Summary

We present a paradigm-shifting drug discovery strategy that transforms counterintuitive insights from a detrimental antibody into targeted therapeutics for both sepsis and rheumatoid arthritis.

## INTRODUCTION

Sepsis, a life-threatening acute inflammatory syndrome, accounts for nearly 20% of global deaths ^1^ and imposes a substantial economic burden, exceeding $62 billion annually in the U.S. alone. Its complex pathogenesis is partly driven by dysregulated innate immune responses, initiated by early proinflammatory cytokines (e.g., tumor necrosis factor, TNF) ^2,3^ and subsequently sustained by late-acting danger-associated molecular patterns (DAMPs) such as high mobility group box 1 (HMGB1) ^4,5^ and inducible procathepsin L (pCTS-L) ^6,7^. In contrast, rheumatoid arthritis (RA) is a chronic autoimmune disease affecting 0.5–1% of the global population ^8^, characterized by persistent synovial inflammation and progressive joint destruction. Despite their distinct etiologies and clinical manifestations, both conditions converge on a common immunopathological mechanism: the dysregulated release of key pro-inflammatory mediators, including cytokines [e.g., TNF, interleukin-1 (IL-1), IL-6] and DAMPs like HMGB1 ^4,5,9–15^. Despite numerous clinical trial failures in sepsis, pre-clinical insights into pathogenic cytokines have facilitated the development of highly effective anti-TNF monoclonal antibodies (mAbs) like infliximab, etanercept, and adalimumab, as cornerstone therapies for RA ^16–20^.

Our previous work, spanning two decades, established HMGB1 as a pivotal inflammatory mediator in both lethal sepsis ^4,5,21–23^ and RA ^11–15^. Upon binding to cell surface receptors such as Toll-like Receptor 4 (TLR4) ^24,25^ or the Receptor for Advanced Glycation End products (RAGE) ^26^, extracellular HMGB1 either induces cytokines/chemokines or, if internalized through RAGE-mediated endocytosis, triggers macrophage pyroptosis ^22,27^. As a highly charged protein, HMGB1 interacts with various endogenous proteins, including haptoglobin ^28^, C1q ^29^, and tetranectin (TN) ^30^, triggering anti-inflammatory responses via distinct signaling pathways. In contrast, cathepsin L (*Ctsl*) is highly inducible in monocytes/macrophages and synovial fibroblasts by bacterial endotoxins (e.g., lipopolysaccharides, LPS) and endogenous cytokines (e.g., IFN-γ, IL-6, and serum amyloid A, SAA) ^6,31–36^. Its precursor, procathepsin L (pCTS-L), can be secreted extracellularly by activated innate immune cells, functioning as a late-acting mediator in lethal sepsis ^6,7,37–39^. Consequently, pCTS-L levels are significantly elevated in the serum and/or synovial fluid of patients with sepsis ^30^ or RA ^40–42^. In RA animal models, *Ctsl* mRNA and CTS-L protein levels are elevated in synovial tissues, particularly within the sublining layer (comprising synovial lining cells and fibroblasts) and perivascular infiltrates (macrophages) ^43,44^. These CTS-L-rich macrophages may directly contribute to subchondral bone erosion, a hallmark of chronic RA ^45^, as genetic depletion or pharmacological suppression of *Ctsl* expression attenuated antigen-induced arthritis ^46^ and cartilage destruction in mice ^47^. However, it was unknown whether HMGB1 could induce pCTS-L expression and secretion in human immune cells, or if pharmacological suppression of pCTS-L expression or extracellular activity could offer protection against sepsis and RA in preclinical settings.

Physiologically, TN circulates abundantly (8–12 μg/ml) in healthy individuals ^48^, but its levels decrease markedly in patients with sepsis ^30^, heart failure ^49,50^, or RA ^51,52^. Genetic depletion of TN results in thoracic spine curvature ^53^, impaired motor function ^54^, deficient wound healing ^55,56^, and increased susceptibility to sepsis ^30^, underscoring TN’s critical importance in health ^57^. Human TN comprises a heparin binding region ^58^, an oligomerization α-helical segment, and a carbohydrate recognition domain (CRD). The CRD domain facilitates binding to multiple proteins, including plasminogen ^59,60^, apolipoprotein A1 ^61^, hepatocyte growth factor (HGF), tissue-type plasminogen activator (t-PA) ^62^, and HMGB1 ^30^. Specifically, a detrimental TN/HMGB1 interaction promotes cellular uptake, leading to macrophage pyroptosis and immunosuppression that may compromise microbial eradication ^30^. Intriguingly, some TN domain-specific mAbs reduced septic lethality ^30,63^; whereas others targeting a distinct epitope paradoxically increased it ^30^. This led us to hypothesize that the epitope of a detrimental anti-TN mAb could be inversely developed as a therapy for inflammatory diseases such as sepsis and RA.

Despite therapeutic advances, urgent needs persist for novel sepsis and RA therapies, as both diseases involve diverse immune pathways and heterogeneous patient populations ^10^. Although anti-TNF therapies have revolutionized RA treatment ^16–18^, they offer only partial efficacy for some patients ^64^, and adversely increase susceptibility to infections ^65^. Patients may also develop tolerance ^66^ or anti-drug antibodies over time ^67^, progressively diminishing the long-term effectiveness of these therapies. Given these limitations, we explored the therapeutic efficacy of an innovative TN-derived P2–1 peptide and a pCTS-L-neutralizing mAb. Here, we present compelling evidence for the therapeutic potential of a TN-derived P2–1 peptide (derived from the epitope of a detrimental mAb9), as well as a pCTS-L-neutralizing mAb2, in animal models of these two inflammatory diseases.

## Results

### Mapping the Epitope of a Detrimental Anti-TN Antibody.

**Figure 1.**
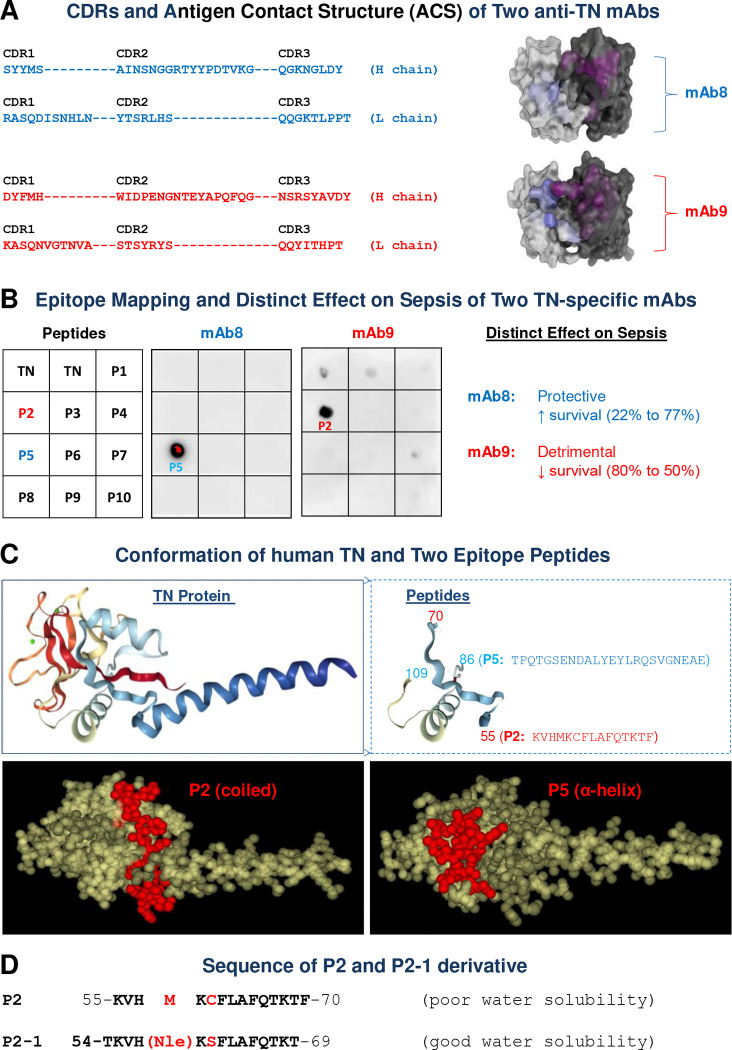
Identification and engineering of P2–1 as an epitope peptide from the detrimental anti-TN mAb (mAb9). **A)** Complementarity Determining Regions (CDRs) and predicted Antigen Contact Structure (ACS) for protective (mAb8) and detrimental (mAb9) anti-TN mAbs. Note the “cave-like” shape of mAb8’s ACS and the “groove-like” conformation of mAb9’s ACS. **B)** Epitope mapping of mAb8 and mAb9 against a panel of TN peptides (P1-P10) via dot blot analysis. While mAb8 binds P5, mAb9 exclusively targets P2, correlating with their previously reported divergent effects on septic survival. **C)** Predicted conformation and spatial arrangement of P2 and P5 epitopes on human TN. Ribbon (Top) and space-fill model (Bottom) models illustrate that P2 and P5 as distinct, accessible epitopes on the TN protein surface, consistent with the predicted ACS. **D)** Amino acid sequence of the P2 epitope and its P2–1 derivative. P2–1 was engineered for enhanced solubility and reduced oxidative susceptibility via specific substitutions: Met (M) to Norleucine (Nle), and Cysteine (C) to Serine (S).

We previously reported that certain anti-TN mAbs, such as mAb8, protected mice against sepsis, while others, exemplified by mAb9, paradoxically exacerbated lethality ^30^, suggesting distinct functional epitopes. To explore the therapeutic potential of this “detrimental” epitope, we determined the amino acid sequence of mAb8 and mAb9 and computationally predicted their Antigen Contact Structures (ACS) (Fig 1A). The ACS of mAb8 presented a “cave-like” shape, suitable for a protruding antigen epitope; whereas mAb9’s ACS exhibited a “groove-like” conformation, implying linear epitope binding. Systematic dot-blotting with ten synthesized TN peptides (P1-P10, fig. S1A, S1B) revealed that mAb8 targeted P5, while mAb9 exclusively interacted with P2 (Fig. 1B). Structural modeling further confirmed P2 and P5 as spatially separated (Fig. 1C, **Top Panel**) and accessible (Fig. 1C, **Bottom Panel**) epitopes on the TN protein surface, consistent with the predicted ACS (Fig. 1A). This structural validation reinforced that targeting different TN epitopes leads to divergent functional outcomes. To enhance therapeutic applicability, we engineered P2–1 for enhanced solubility and reduced oxidative susceptibility via specific substitutions: Methionine (M) to Norleucine (Nle) and Cysteine (C) to Serine (S) (Fig. 1D).

### A TN’s C-terminal CRD Retained Protective Properties in Sepsis.

Given TN’s diverse functional domains (fig. S1A) and our P2 epitope findings, we sought to delineate its core protective mechanism. We generated a recombinant TN mutant (ΔTN, residues 45–181) lacking the N-terminal heparin binding and α-helix trimerization regions (fig. S1C), and evaluated its therapeutic potential in the cecal ligation and puncture (CLP) model of sepsis. As shown in fig. S1D, ΔTN conferred significant protection against lethal sepsis, comparable to full-length TN ^30^, indicating that TN’s protective functionality was largely retained within its C-terminal CRD, which notably encompasses the P2 epitope (residues 55–70, fig. S1A, S1B). This finding suggested that the oligomeric state, mediated by the N-terminal trimerization region, might not be essential for TN’s protective effects. To pinpoint the minimal active region for these benefits, we explored the protective capacities of ten synthetic peptides from the human TN CRD (P1-P10, fig. S1B), including P2, in the CLP sepsis model.

### P2 and P2–1 Derivative Conferred Significant Protection Against Lethal Sepsis.

**Figure 2.**
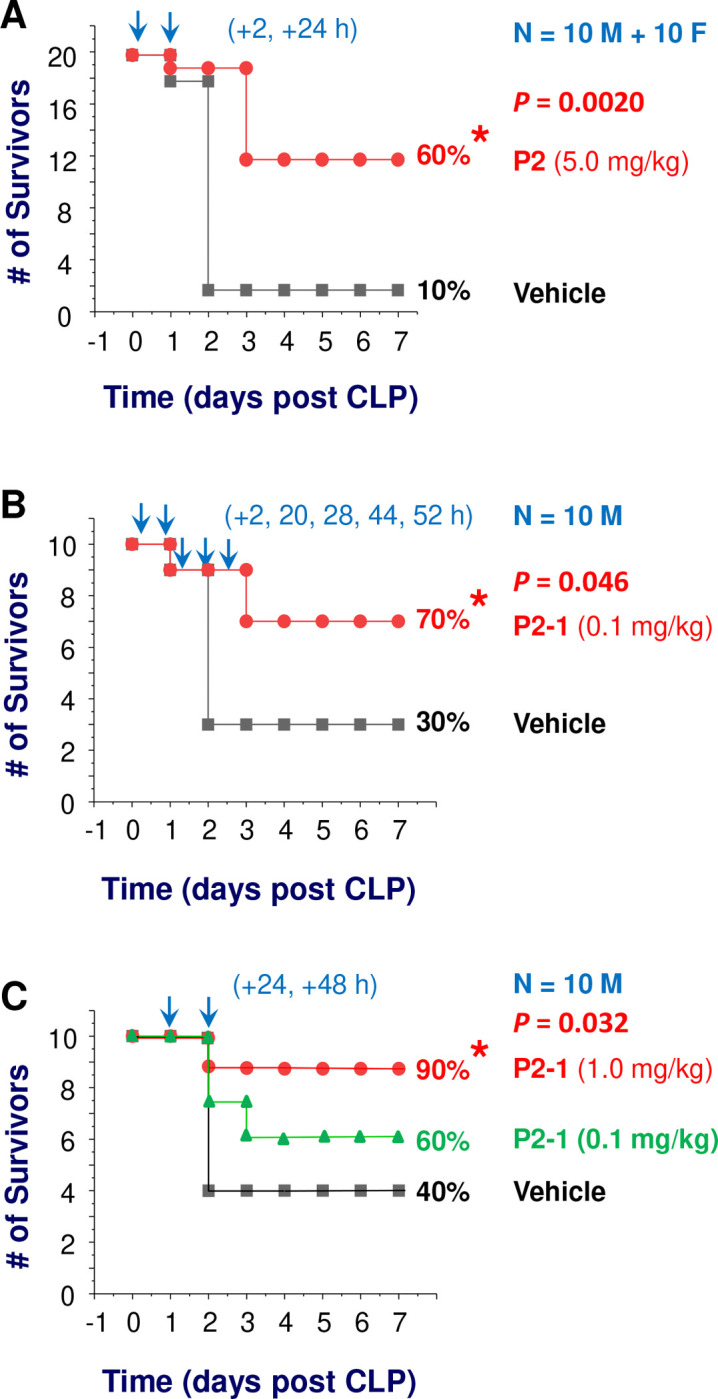
P2 and its P2–1 derivative conferred significant protection against lethal sepsis. **A)** Repetitive administration of P2 peptide (5.0 mg/kg at 2 h and 24 h post-CLP) significantly increased animal survival in male (“M”) and female (“F”) mice. *, *P <* 0.05 vs. vehicle group. **B)** P2–1 derivative (0.1 mg/kg, administered at 2 h, 20 h, 28 h, 44 h, and 52 h post-CLP) also conferred significant protection against lethal sepsis. *, *P* < 0.05 vs. control group. **C)** Delayed administration of P2–1 (1.0 mg/kg at 24 h and 48 h post-CLP) rescued mice from lethal sepsis. *, *P* < 0.05 vs. vehicle group.

The identification of P2 epitope as the binding site for mAb9, which paradoxically reduced septic survival ^30^, presented a compelling therapeutic opportunity. We evaluated the therapeutic efficacy of both P2 and its water-soluble derivative, P2–1, in CLP-induced sepsis. Repetitive administration of P2 (5.0 mg/kg twice at 2 h and 24 h post-CLP) significantly increased survival rates in both male and female mice (Fig. 2A). Similarly, P2–1, at a lower dose (0.1 mg/kg) and multiple time points (e.g., 2 h, 20 h, 28 h, 44 h, and 52 h post-CLP), also conferred significant protection against sepsis (Fig. 2B), indicating that P2–1 retained P2’s efficacy with improved pharmacological properties. Crucially, P2–1 provided potent protection against lethal sepsis even with delayed therapeutic administration (24 h and 48 h post-CLP) at a dose (1.0 mg/kg) approximately 5-fold lower than P2 (Fig. 2C). The ability of P2–1 to provide protection in a delayed therapeutic setting is particularly significant for clinical sepsis, where early intervention is often challenging.

**Figure 3.**
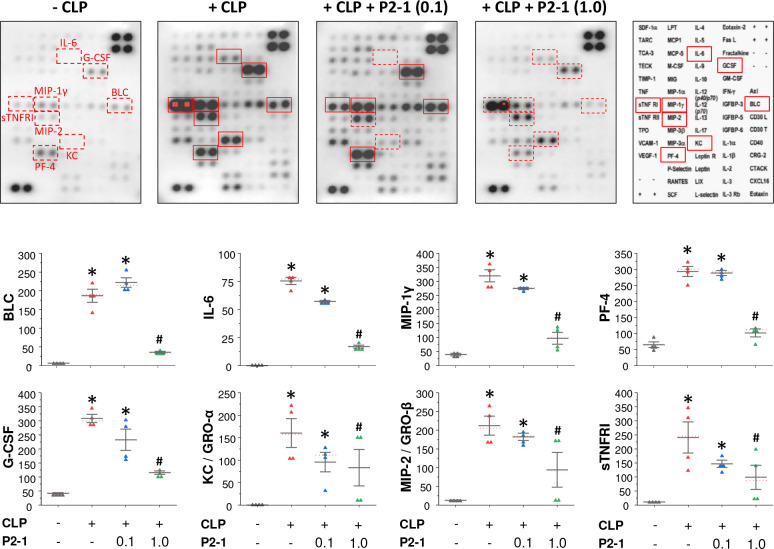
P2–1 significantly attenuated sepsis-induced systemic inflammation. Balb/C mice subjected to CLP-induced sepsis received P2–1 (0.1 or 1.0 mg/kg) intraperitoneally twice, at 2 h and 20 h post-CLP. At 24 h post-CLP, animals were sacrificed to harvest blood for cytokine and chemokine measurements using Cytokine Antibody Arrays. *, *P* < 0.05 vs. negative control (“− CLP”); #, *P* < 0.05 vs. positive control (“+ CLP”) group, non-parametric Kruskal-Wallis ANOVA test.

To investigate P2–1’s protective mechanisms, we assessed its effects on sepsis-induced systemic inflammation. Intraperitoneal P2–1 administration significantly reduced sepsis-induced systemic accumulation of BLC/CXCL13, granulocyte colony stimulating factor (G-CSF), interleukin-6 (IL-6), keratinocytes-derived chemokine (KC)/GRO-α/CXCL1, monocyte chemoattractant protein-1 (MCP-1)/CCL2, macrophage inflammatory protein 1γ (MIP-1γ)/CCL9/10, MIP-2/GRO-β/CXCL3, and soluble tumor necrosis factor receptor I (sTNFRI) (Fig. 3). This comprehensive reduction in a broad panel of pro-inflammatory cytokines and chemokines indicates that P2–1 confers protection against sepsis by effectively mitigating systemic inflammation, consistent with its effect on key surrogate biomarkers of experimental ^68,69^ and clinical sepsis ^70^.

### P2–1 Conferred a Dose-Dependent Protection Against Collagen Antibody-Induced Arthritis (CAIA).

**Figure 4.**
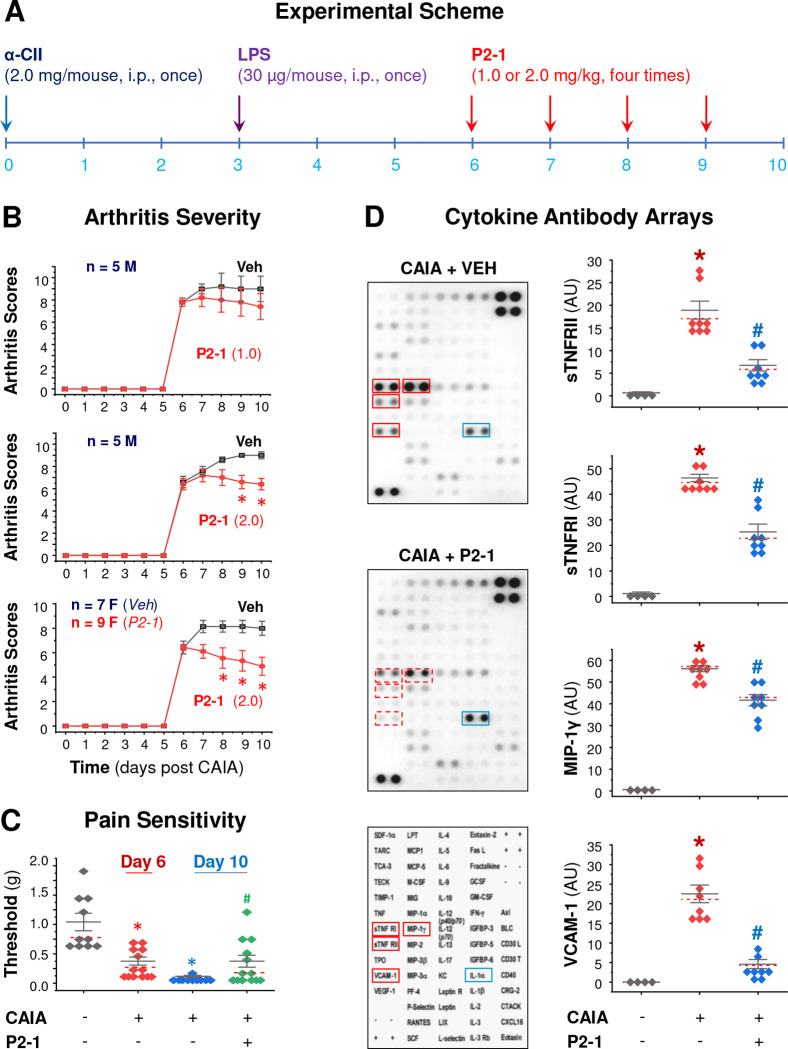
P2–1 conferred a dose-dependent protection against collagen antibody-induced arthritis (CAIA). **A)** Experimental scheme for the CAIA induction and P2–1 therapeutic intervention in Balb/C mice. **B)** P2–1 significantly attenuated CAIA-induced arthritis severity in both male (“M”) and female (“F”) mice. *, *P* < 0.05 vs. saline vehicle (“Veh”) group, non-parametric Kruskal-Wallis ANOVA test. **C)** P2–1 significantly reduced CAIA-induced pain sensitivity by partially restoring paw mechanical withdrawal thresholds. *, *P* < 0.05 vs. non-arthritic negative control (“− CAIA”). #, *P* < 0.05 vs. vehicle (“Veh”)-treated positive control (“+ CAIA”) on the same day. **D)** P2–1 reduced CAIA-induced joint tissue inflammation. Cytokine antibody arrays of joint tissue lysates (harvested Day 10) show reduced levels of sTNFRI, sTNFRII, MIP-1γ, and VCAM-1 in P2–1-treated mice. *P < 0.05 vs. non-arthritic negative control (“− CAIA”); #, *P* < 0.05 vs. vehicle (“Veh”)-treated CAIA control (“+ CAIA”), non-parametric Kruskal-Wallis ANOVA test.

Extending our success in experimental sepsis, we further evaluated P2–1’s efficacy in the collagen antibody-induced arthritis (CAIA) model. CAIA is a clinically relevant animal model that faithfully recapitulates many characteristics of human RA, including rapid onset of polyarthritis, joint inflammation, cartilage degradation, and bone erosion. Furthermore, microbial infections can often trigger inflammatory responses in the joints, driving the onset, progression, and outcomes of RA ^20,71–73^. As depicted in Fig. 4A, CAIA was induced with anti-collagen II mAbs (α-CII) on Day 0, followed by LPS on Day 3 to activate inflammatory responses and synchronize disease onset. Representing a therapeutic intervention in established disease, P2–1 administration began on Day 6 post-α-CII challenge and continued daily for four consecutive days. Remarkably, P2–1 dose-dependently and significantly attenuated CAIA-induced arthritis severity in both male (“M”) and female (“F”) mice (Fig. 4B), highlighting its broad applicability across sexes.

As RA is also characterized by significant pain responses, a major driver of patient morbidity and reduced quality of life, we measured mechanical allodynia (pain sensitivity) using Von Frey filaments. As shown in Fig. 4C, P2–1 significantly reduced CAIA-induced pain sensitivity by partially restoring withdrawal thresholds to mechanical stimuli. Furthermore, P2–1 significantly reduced CAIA-induced joint tissue inflammation (Fig. 4D; fig. S2), evidenced by the significant reduction of soluble TNF receptors (sTNFRI and sTNFRII), chemokine MIP-1γ/CCL9/10, and vascular cell adhesion molecule 1 (VCAM-1) in the joint tissue. These findings collectively suggested that P2–1 exerted its protective effects by dampening the inflammatory milieu within the affected joints, thereby ameliorating both clinical disease progression and pain.

### P2–1 Interacted with HMGB1 to Selectively Block HMGB1-Induced *Ctsl* mRNA Upregulation and pCTS-L Secretion.

**Figure 5.**
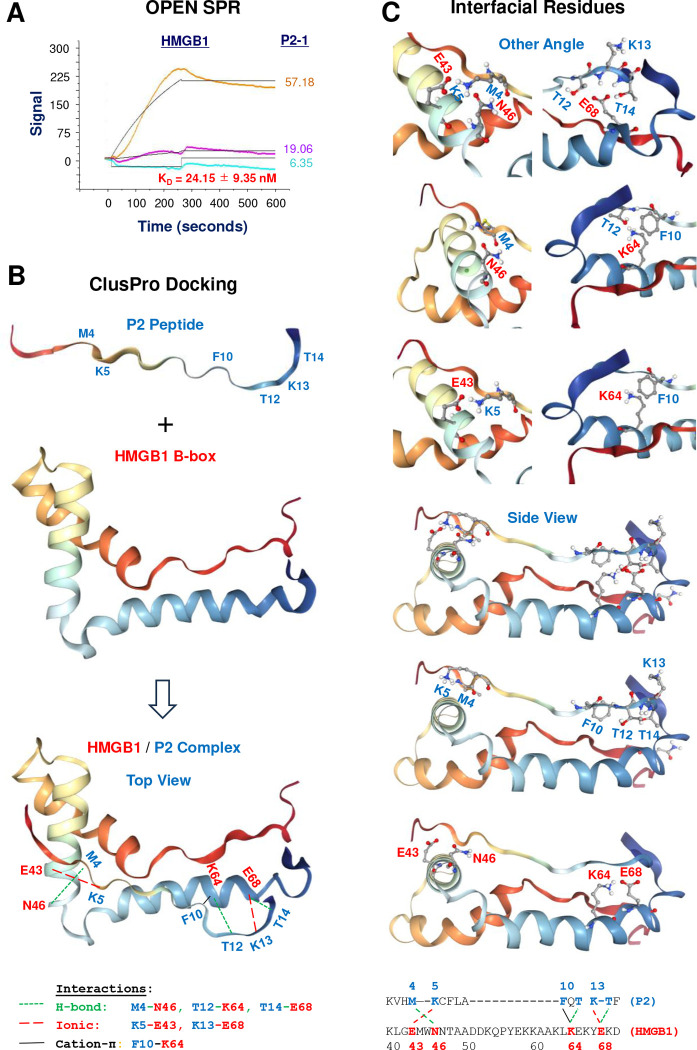
P2–1 interacted with HMGB1 with high affinity and selectivity. **A)** SPR analysis of P2–1/HMGB1 interaction, revealing dose-dependent binding of P2–1 to immobilized HMGB1 with an estimated K_D_ of 25 nM (Mean ± SEM, n = 2 independent experiments). **B)** ClusPro protein-protein docking of the HMGB1/P2 complex, highlighting key hydrogen bonds, ionic, and cation-π interactions at the binding interface. **C)** Detailed views of interfacial residues in the HMGB1-P2 complex from various angles, illustrating the specific amino acid interactions.

Prompted by P2–1’s consistent therapeutic efficacy in both sepsis and CAIA, we investigated its underlying molecular mechanism. We hypothesized that P2–1, derived from its precursor TN, exerts beneficial effects by directly binding pathogenic HMGB1 to modulate its detrimental functions. Surface Plasmon Resonance (SPR) confirmed P2–1’s high-affinity binding to HMGB1, with an equilibrium dissociation constant (K_D_) of approximately 25 nM (Fig. 5A). To gain structural insights, we predicted the P2 peptide structure (fig. S3A) and performed protein-protein docking with the human HMGB1 B-box domain (residues 89–167). This docking model (fig. S3B, Fig. 5B) revealed that P2 directly interacted with the HMGB1 B-box (fig. S3C), a region critical for HMGB1’s pro-inflammatory functions ^24^. Notably, key residues involved in P2 interaction (e.g., E43, N46, K64, and E68 of HMGB1 B-box, Fig. 5B, 5C; or E131, N134, K152, and E156 of HMGB1, fig. S3C) were spatially distinct from its TLR4-binding domain (residues 89–108, fig. S3C), but partially overlapped with the RAGE-binding region (residues 150–183, fig. S3C) ^74,75^. This spatial relationship suggests that P2–1, by binding to this P2-interacting region of HMGB1, can selectively influence RAGE-dependent HMGB1 activities (e.g., endocytosis and macrophage pyroptosis) without impacting its TLR4-dependent functions (e.g., induction of cytokines and chemokines).

**Figure 6.**
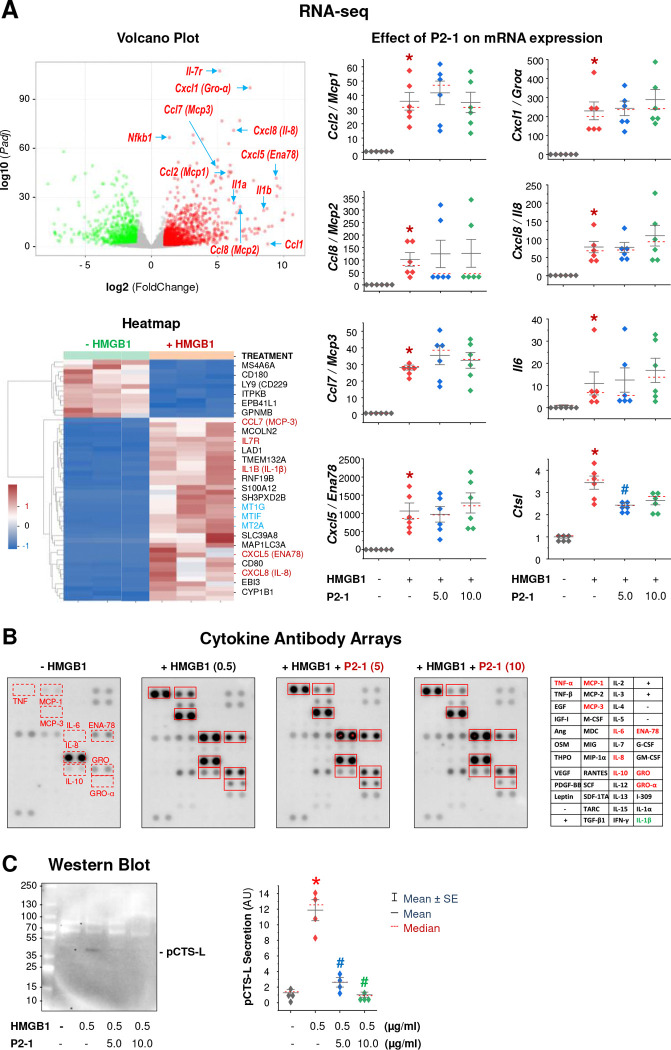
P2–1 selectively inhibited HMGB1-induced *Ctsl* expression and pCTS-L secretion in human immune cells. **A)** RNA-seq analysis of gene expression in human PBMCs from three independent donors, stimulated with HMGB1 in the absence or presence of P2–1. Quantitative analysis of mRNA expression demonstrated that P2–1 significantly reduced HMGB1-induced *Ctsl* upregulation, while not significantly affecting the expression of other HMGB1-induced cytokines and chemokines. **B)** Cytokine Antibody Array analysis of HMGB1-induced cytokine and chemokine secretion in human PBMCs. P2–1 (5 or 10 μg/ml) did not affect the secretion of these HMGB1-induced cytokines and chemokines. **C)** Western blotting analysis of HMGB1-induced pCTS-L secretion in human PBMCs. P2–1 dose-dependently abrogated HMGB1-induced pCTS-L secretion (n = 4 independent experiments). *, P < 0.05 vs. negative control (“− HMGB1”); #, *P* < 0.05 vs. positive control (“+ HMGB1”), non-parametric Kruskal-Wallis ANOVA test.

To identify P2–1’s downstream targets, we performed RNA-seq analysis on human peripheral blood mononuclear cells (PBMCs) stimulated with HMGB1, with or without P2–1 co-treatment. The volcano plot and heatmap (Fig. 6A) revealed that HMGB1 stimulation profoundly upregulated many inflammatory cytokines (e.g., IL-1β) and chemokines [e.g., CXCL5/ENA78, CCL7/MCP-3, and CXCL8/IL-8] (**Data File S1**), validating HMGB1’s critical role as an inflammatory mediator of sepsis and RA ^7,75,76^. However, P2–1 did not broadly reverse HMGB1-induced transcriptional changes for most genes (fig. S4A, S4B), including highly upregulated inflammatory cytokines (e.g., *Il6*) and chemokines (e.g., *Ccl2/Mcp1, Ccl8/Mcp2, Ccl7/Mcp3, Cxcl5/Ena78, Cxcl1/Groα, Cxcl8/Il8*) (Fig. 6A, **Right Panels**; **Data File S1**). In a sharp contrast, P2–1 treatment moderately but significantly reduced HMGB1-induced *Ctsl* mRNA upregulation, decreasing from 3.4 ± 0.3-fold (in the HMGB1-only group) to 2.4 ± 0.1-fold (in the HMGB1 + P2–1” group) (Fig. 6A, **Right Panel; Data File S1**).

To validate these RNA-seq findings, we quantified HMGB1-induced cytokine/chemokine protein levels using Cytokine Antibody Arrays. Consistent with the RNA-seq data, HMGB1 stimulation significantly increased protein levels of various cytokines (e.g., TNF, IL-6, and IL-10) and chemokines (e.g., CXCL5/ENA78, CXCL1/GRO-α, CXCL8/IL-8, CCL2/MCP-1, and CCL7/MCP-3) in human PBMCs (Fig. 6B), confirming its potent immunomodulating activities ^76^. However, P2–1 did not broadly inhibit the secretion of these HMGB1-induced cytokines and chemokines (Fig. 6B, fig. S5), underscoring the selectivity of its action. This selectivity aligns with previous findings that TN, P2–1’s precursor, similarly did not affect HMGB1-induced cytokines (e.g., IL-6) and chemokines (e.g., MCP-1) ^30^. In stark contrast, Western blot analysis revealed a striking and dose-dependent suppression of HMGB1-induced pCTS-L secretion by P2–1 in human PBMCs (Fig. 6C, fig. S6). This finding, coupled with our RNA-seq results, collectively demonstrate that P2–1 selectively inhibits HMGB1-induced *Ctsl* mRNA upregulation and pCTS-L secretion, implicating a pathogenic role for the HMGB1-pCTS-L axis in sepsis and RA.

### A pCTS-L-Neutralizing mAb Conferred Protection against Collagen Antibody-Induced Arthritis.

**Figure 7.**
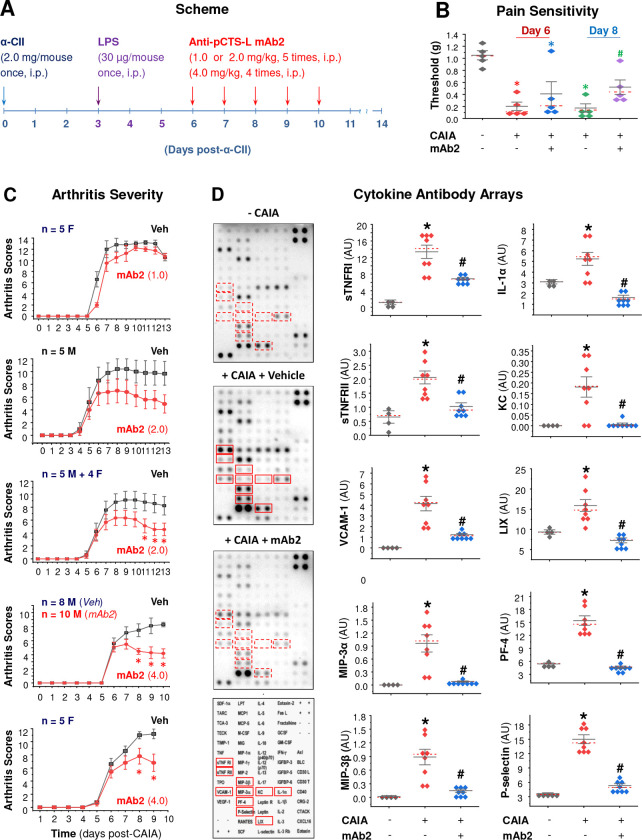
A pCTS-L-neutralizing mAb (mAb2) conferred dose-dependent protection against collagen antibody-induced arthritis (CAIA). **A)** Experimental scheme for CAIA induction and pCTS-L-neutralizing mAb2 therapeutic intervention in Balb/C mice. **B)** mAb2 significantly reduced CAIA-induced pain sensitivity by partially restoring paw mechanical withdrawal thresholds. *, *P* < 0.05 versus non-arthritic negative control (“− CAIA”). #, *P* < 0.05 vs. vehicle (“Veh”)-treated positive control (“+ CAIA”) on the same day, non-parametric Kruskal-Wallis ANOVA test. **C)** mAb2 significantly attenuated CAIA-induced arthritis severity in both male (“M”) and female (“F”) mice. *, *P* < 0.05 vs. vehicle control group, non-parametric Kruskal-Wallis ANOVA test. **D)** mAb2 reduced CAIA-induced joint tissue inflammation. *, *P* < 0.05 vs. non-arthritic negative control (“− CAIA”); #, *P* < 0.05 vs. vehicle (“Veh”)-treated CAIA control (“+ CAIA”), non-parametric Kruskal-Wallis ANOVA test.

Given pCTS-L’s pathogenic role in sepsis ^6,7,37–39^ and its elevation in experimental animals ^43–45^ and patients with RA ^40–42^, we evaluated the therapeutic potential of a pCTS-L-neutralizing mAb in the CAIA model of RA (Fig. 7A). Administration of the pCTS-L-neutralizing mAb2 significantly mitigated CAIA-induced pain sensitivity (Fig. 7B), evidenced by a partial normalization of withdrawal thresholds to mechanical stimulation. Consistently, mAb2 dose-dependently and significantly attenuated CAIA-induced arthritis severity in both male (“M”) and female (“F”) mice (Fig. 7C), mirroring the broad efficacy of P2–1, a selective pCTS-L inhibitor, in preclinical settings. These symptomatic and clinical benefits correlated with significant reductions in various pro-inflammatory cytokines (e.g., IL-1α), chemokines (e.g., CCL20/MIP-3α, CCL19/MIP-3β, CXCL1/KC/GRO-α, and LIX/ENA-78/CXCL5), soluble TNF receptors (sTNFRI/II), and adhesion molecules (e.g., VCAM-1 and P-selection) within the joint microenvironment (Fig. 7D, fig. S7). Collectively, these results demonstrate that the pCTS-L-neutralizing mAb2 provides robust therapeutic efficacy in the CAIA model, successfully ameliorating both clinical manifestations of arthritis and associated pain by suppressing the local inflammatory cascade within the inflamed joints.

### Histopathological Evidence: pCTS-L-neutralizing mAb Protected against Arthritis Progression and Joint Destruction

**Figure 8.**
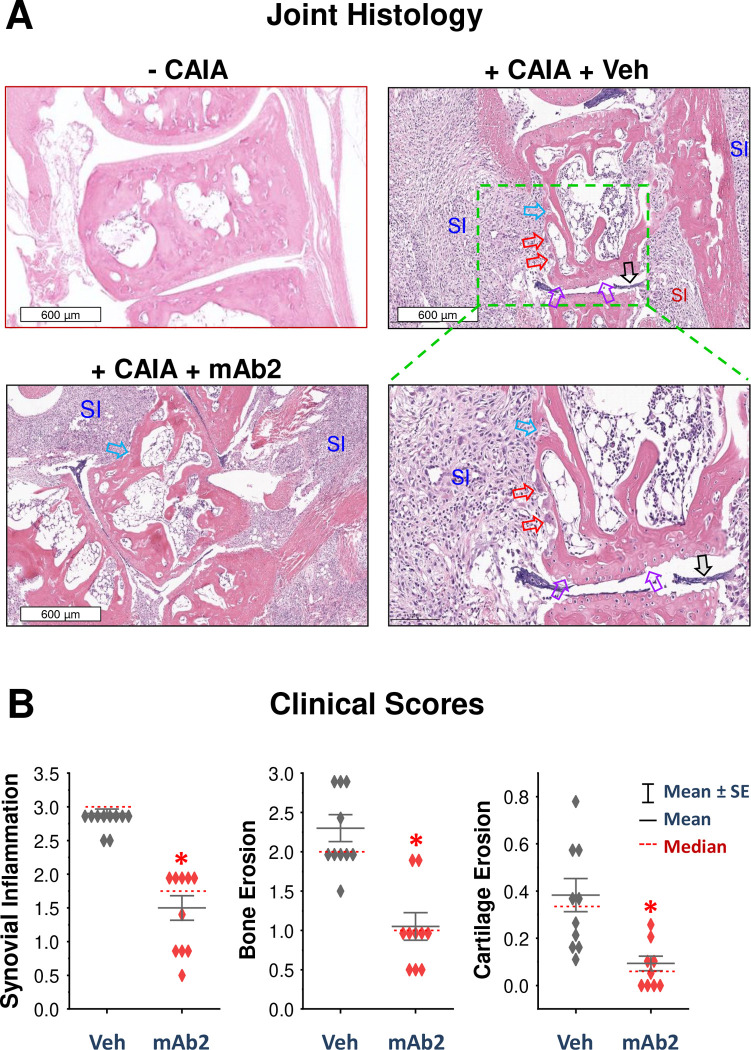
A pCTS-L-neutralizing mAb reduced ankle joint inflammation and structural damage in CAIA mice. **A)** Representative histological analysis of H&E-stained ankle joints from normal (“ −CAIA”), saline vehicle-treated CAIA (“+ CAIA + Veh”), and pCTS-L-neutralizing mAb2-treated CAIA (“+ CAIA + mAb2”) mice. Vehicle-treated CAIA mice exhibited severe synovial joint inflammation (SI) characterized by extensive inflammatory cell infiltration, pronounced synoviocyte hyperplasia, thickening of synovial lining and sublining, and erosion of bone (**blue arrow**) and cartilage (**purple arrows**). The expanded synovial lining, combined with dense inflammatory infiltrates and activated fibroblasts in the sublining, collectively formed highly aggressive, tumor-like structures called “pannus” (**black arrows**). Pannus invades the adjacent cartilage and bone, resulting in osteoclast clustering on the bone surface (**red arrows**) and subsequent bone erosion. Note that mAb2 treatment profoundly attenuated these pathologies, preserving overall joint architecture. **B)** Quantitative histological assessment of synovial inflammation, bone erosion, and cartilage erosion in mAb2-treated CAIA mice compared to vehicle controls. *, *P* < 0.05 vs. vehicle control, non-parametric Kruskal-Wallis ANOVA test.

Complementing these pre-clinical findings, histological analysis of ankle joints from vehicle-treated CAIA animals revealed profound synovial joint inflammation (SI), marked by extensive inflammatory cell infiltration, pronounced synoviocyte hyperplasia, and substantial thickening of the synovial lining and sublining (Fig. 8A). The expanded synovial lining, combined with dense inflammatory infiltrates and activated fibroblasts in the sublining, collectively formed highly aggressive, tumor-like structures known as “pannus” (Fig. 8A, **black arrow**). This pannus could relentlessly invade and degrade adjacent cartilage and bone, promoting osteoclast clustering on the bone surface (**red arrows**) and subsequent erosion of bone (**blue arrow**) and cartilage (**purple arrows**). In striking contrast, pCTS-L-neutralizing mAb2 treatment profoundly attenuated these severe histological pathologies. Joints from mAb2-treated animals exhibited substantially reduced inflammatory cell infiltration, diminished synovial hyperplasia and thickening, and critically, amelioration of both bone and cartilage erosion (Fig. 8A). Quantitative histological scoring of synovial inflammation (SI), bone erosion, and cartilage erosion further confirmed statistically significant reductions in all measured parameters with mAb2 treatment compared to vehicle controls (Fig. 8B). The mAb2’s ability to prevent both inflammation and structural damage reinforces pCTS-L as a promising therapeutic target for disease-modifying intervention in RA.

## Discussion

Sepsis and RA are distinct yet mechanistically related inflammatory conditions commonly driven by dysregulated innate immune responses and excessive cytokine production. Despite extensive research, effective sepsis therapies remain elusive, and existing RA treatments often suffer from limited efficacy and significant side effects. Our study introduces a paradigm-shifting drug discovery strategy: repurposing an epitope derived from a “detrimental” anti-TN antibody ^30^ into P2–1, a targeted therapy for both sepsis and RA. This counterintuitive approach originated from our observation that different anti-TN antibodies, targeting different epitopes, yielded divergent functional outcomes ^30^. We hypothesized that the P2 epitope itself, when administered as a standalone peptide, would serve as a decoy, competing with TN for HMGB1 binding and thereby disrupting its detrimental functions (fig. S8).

Our study demonstrates that P2–1 directly binds HMGB1, a critical mediator in both sepsis ^5,21–23^ and RA ^11–15^, through specific interactions with key residues (E131, N134, K152, and E156) of HMGB1’s B-box domain. Crucially, these P2-binding sites are spatially separated from HMGB1’s TLR4-binding region (residues 89–108) but partially overlap with the RAGE-binding domain (residues 150–183). This unique spatial geometry implies that P2 and P2–1 could potentially influence RAGE-dependent HMGB1 activities (e.g., endocytosis and macrophage pyroptosis) ^22,27^ without impacting its TLR4-dependent functions (e.g., induction of cytokines and chemokines) ^24,25^. Indeed, P2–1 did not broadly suppress HMGB1-induced cytokines and chemokines, but selectively inhibited HMGB1-mediated *Ctsl* mRNA upregulation and pCTS-L secretion, thereby serving as a precise strategy to target the HMGB1-pCTS-L axis (fig. S8). This approach not only offers a sophisticated strategy to modulate pervasive inflammatory pathways but also underscores the value of re-evaluating initial non-efficacious (negative) biological observations for untapped (hidden) therapeutic potential in drug discovery.

The significance of this selective mechanism was further underscored by our direct validation of pCTS-L as a therapeutic target. While pCTS-L’s role as a late-acting mediator of lethal sepsis was recently identified ^6,7^, its direct involvement and therapeutic potential in RA were less explored. Here, we established a mechanistic link between HMGB1 and pCTS-L by demonstrating that HMGB1, a critical mediator of sepsis ^5,21–23^ and RA ^11–15^, induced pCTS-L expression and secretion in human immune cells (fig. S8). Furthermore, a pCTS-L-neutralizing antibody (mAb2) significantly attenuated clinical arthritis and pain in the CAIA model, profoundly ameliorating joint inflammation, cartilage destruction, and bone erosion. The ability of a neutralizing antibody to mitigate RA progression positions pCTS-L as a highly promising and novel therapeutic target, especially offering an alternative pathway beyond current anti-TNF therapies. Our findings align with previous observations of elevated CTS-L levels in animal models ^43–45^ and RA patients ^40–42^, and agree with the protective effects of genetic or pharmacological *Ctsl* suppression in experimental arthritis ^46,47^.

A notable aspect of this research is the successful translation of a therapeutic strategy from acute sepsis to chronic RA, diseases that share common inflammatory pathways involving key mediators such as TNF, HMGB1, and pCTS-L (fig. S8). The consistent efficacy of P2–1 in both models, even with delayed therapeutic administration, underscores its potential as a selective anti-inflammatory agent for the HMGB1-pCTS-L axis. The ability of P2–1 to reduce systemic inflammatory mediators in sepsis (e.g., IL-6, KC/GRO-α/CXCL1, MIP-2/GRO-β/CXCL3) and local joint inflammation in RA (e.g., sTNFRI, sTNFRII, MIP-1γ/CCL9/10, VCAM-1) reinforces its immunomodulatory effects against key inflammatory mediators within the HMGB1-pCTS-L axis (fig. S8). This “cross-disease” efficacy suggests that modulation of the HMGB1-pCTS-L axis represents a central, critical pathway in generalized inflammation, making it an attractive target for various inflammatory disorders.

Despite these promising findings, certain limitations warrant discussion. Our reliance on preclinical animal models of sepsis and RA necessitates careful consideration of species-specific differences in immune responses during clinical translation. While we elucidated a key mechanistic pathway involving P2–1, HMGB1, and pCTS-L, the full spectrum of P2–1’s interactions and downstream effects requires further exploration. The selective suppression of HMGB1-induced *Ctsl* mRNA upregulation by P2–1, without broadly reversing other HMGB1-induced cytokines/chemokines, highlights its specificity but also calls for deeper investigation into its precise targets. Finally, future work will need to address the long-term safety, pharmacokinetics, and pharmacodynamics of P2–1 in larger animal models and ultimately, in human clinical trials.

In conclusion, our study unveils a paradigm-shifting drug discovery strategy that transforms counterintuitive insights from a detrimental antibody into powerful and targeted therapeutics. This approach yielded P2–1 as a novel therapy that conferred significant protection against both sepsis and RA, even when administered therapeutically after disease onset. Mechanistically, P2–1 binds HMGB1 to selectively suppress HMGB1-induced *Ctsl* mRNA upregulation and pCTS-L secretion, thereby effectively disrupting the crucial HMGB1-pCTS-L inflammatory axis (fig. S8). Furthermore, our findings establish pCTS-L as a key mediator of inflammatory diseases, validating it as a novel therapeutic target for RA. These findings open exciting new avenues for drug development, underscoring the potential of precisely modulating harmful protein-protein interactions (via decoy peptides or neutralizing antibodies) to achieve therapeutic outcomes in complex inflammatory disorders.

## Materials and Methods

### Study Design

For these pre-clinical studies, animals were randomly assigned to experimental groups and treated with P2, P2–1, or a pCTS-L-neutralizing monoclonal antibody (mAb2) per indicated dosing regiments. Outcomes, including septic survival rates, arthritis severity, pain sensitivity, and joint tissue histology scores, were collected under blinded experimental conditions. Study design and sample sizes for each experiment are detailed in the figure legends. No data, including outlier values, were excluded. Primary data are reported in **Data File S1**. All reagent sources are listed in Table S1.

### Animal Models and Ethics Statement

We adhered to ARRIVE guidelines for minimizing animal numbers and the Minimum Quality Threshold in Pre-Clinical Sepsis Studies (MQTiPSS) ^77^. Practices included randomization, delayed therapeutic interventions ^78^, establishment of criteria for euthanizing moribund animals (e.g., labored breathing, minimized response to touch, and immobility), and administration of fluid resuscitation and antibiotics to septic animals ^79^. This study was approved by the Institutional Animal Care and Use Committee (IACUC) of the Feinstein Institutes for Medical Research (Protocol #2017–027 Term II, August 24, 2020; #2023–002, April 6, 2023; and #2024–0846, October 29, 2024). Male and female Balb/C (8–10 weeks old, 20–25g) were obtained from Jackson Laboratory (Bar Harbor, ME) or Charles River Laboratories (Wilmington, MA), and acclimated for at least 5–7 days, in specific pathogen-free conditions with free access to food and water, prior to experimentation. Animals were housed Experiments were conducted in sex-segregated groups to assess potential sex-specific differences; data were pooled when no significant differences were observed, thereby minimizing animal numbers while ensuring robust conclusions.

### Cecal Ligation and Puncture (CLP) Sepsis Model

Experimental sepsis was induced in Balb/C mice via cecal ligation and puncture (CLP) as previously described ^6,30,37^. Prior to CLP surgery, all animals received buprenorphine once (0.05 mg/kg, subcutaneously) for pain management, as repetitive buprenorphine use in CLP could paradoxically elevate sepsis surrogate markers and increase animal lethality ^80,81^. Briefly, mice were anesthetized intramuscularly with ketamine (75 mg/kg, Henry Schein Animal Health, Dublin, Ohio) and xylazine (10  mg/kg, Sigma-Aldrich) before a midline abdominal incision was made in the lower left abdomen. The cecum was ligated with a 4–0 silk suture at 50% of its length from the distal end, and punctured twice with a 21-gauge needle to induce moderate to severe sepsis. The cecum was returned to the abdominal cavity, and the incision was closed in layers. Approximately 30 min post-CLP, animals received imipenem/cilastatin (0.5 mg/mouse, Primaxin, Merck & Co., Inc.) and 1.0 mL of sterile saline for fluid resuscitation. Animals were randomized to control vehicle and experimental groups, administered P2 or P2–1 intraperitoneally at indicated doses and time points, and monitored for survival rates. To elucidate P2–1’s protective mechanisms, a separate group of Balb/C mice underwent CLP and received P2–1 (1.0 mg/kg) at 2 h and 20 h post-CLP. At 24 h post-CLP, animals were euthanized to collect blood and measure serum cytokines and chemokines using murine Cytokine Antibody Arrays ^30^.

### Collagen Antibody-Induced Arthritis (CAIA) Model

The CAIA model was performed in Balb/C mice (8–10 weeks old) as described previously ^15^. On Day 0, mice received i.p. injection of 2 mg anti-collagen II mAb cocktail (α-II, Chondrex, Inc., Redmond, WA). On Day 3, mice received i.p. injection of 30 μg LPS (*Escherichia coli* O111:B4; Sigma-Aldrich) to activate inflammation and synchronize arthritis onset. Mice were randomized, and therapeutic agents (P2–1 peptide or pCTS-L-neutralizing mAb2) were administered i.p. daily from Day 6 to Day 9. Arthritis severity was scored daily using a clinical scoring system [0 – 4 per paw: 0 = normal; 1 = mild redness and swelling in a single joint type (ankle, wrist, or individual digits); 2 = moderate redness and swelling affecting two joint types; 3 = severe redness and swelling involving across all three joint types; 4 = maximal redness and swelling of the entire paw that obscured joint definition].

### Pain Sensitivity Assessment (Von Frey Filaments)

Mechanical allodynia was assessed using Von Frey filaments (Stoelting Co., Wood Dale, IL) applied to the plantar surface of the hind paws as previously described ^15,82^. Mice were habituated in individual transparent enclosures on an elevated wire mesh platform. Calibrated Von Frey filaments (0.008 g to 2.0 g) were applied for 2–3 seconds or until a withdrawal response. The “up-down” method determined the 50% paw withdrawal threshold, defined as the force required to elicit a withdrawal response in 50% of applications.

### Histopathological Analysis

Ankle joints were harvested from CAIA mice at the end of the study, decalcified in 10% EDTA, and embedded in paraffin. Sections (5 μm) were cut and stained with Hematoxylin and Eosin (H&E) for overall morphology and inflammation. As previously described ^83^, histopathological scoring was performed by a blinded pathologist based on established criteria: synovial inflammation (SI, 0 – 3 scale: 0 = no inflammation, 1 = mild, 2 = moderate, 3 = severe), bone erosion (0 – 3 scale: 0 = no erosion, 1 = mild, 2 = moderate, 3 = severe), and cartilage erosion (0 – 3 scale: 0 = no erosion, 1 = mild, 2 = moderate, 3 = severe). Representative images were captured using a brightfield microscope (Nikon Eclipse Ti2).

### Protein Expression, Purification, and Peptide Synthesis

Recombinant human HMGB1 was expressed in *E. coli* BL21 (DE3) pLysS and purified as described ^4^. Recombinant TN mutant (ΔTN), lacking residues 1–44 (heparin binding and part of the α-helix trimerization domain) was expressed *E. coli* BL21 (DE3) pLysS with a N-histidine tag, and purified to homogeneity following a similar protocol to that for full-length TN ^30^. Briefly, after sonication to disrupt bacteria, ΔTN inclusion bodies were isolated by differential centrifugation following extensive washing in 1 × PBS containing 1% Triton X-100. The inclusion bodies were then solubilized in 8 M urea, and refolded by dialysis in 10 mM Tris buffer (pH 8.0) containing N-lauroylsarcosine. Subsequently, the recombinant ΔTN was subjected to extensive Triton X-114 extractions to remove contaminating endotoxins. P2 peptide (55-KVHMKCFLAFQTKTF-70) and its water-soluble derivative P2–1 [54-TKVH(Nle)KSFLAFQTKT-69] were custom-synthesized (> 95% purity; GenScript, Piscataway, NJ). P2–1 was engineered for enhanced solubility and reduced oxidative susceptibility via minimal amino acid substitutions: Methionine (M) to Norleucine (Nle) and Cysteine (C) to Serine (S). The pCTS-L-neutralizing mAbs (mAb2) and TN-neutralizing mAbs (mAb8 and mAb9) were generated in Balb/C and C57BL/6 mice as previously described ^6,30^.

### Antibody Sequencing and Antigen Contact Structure (ACS) Prediction

Total RNA was extracted from mAb8 and mAb9 hybridoma cells using TRIzol (Invitrogen) and cDNA was synthesized using reverse transcriptase. Variable regions of heavy and light chains were amplified by PCR using degenerate primers and sequenced ^30^. CDR sequences were identified using the Kabat numbering scheme. Computational modeling of the mAb-antigen interaction was performed using ABodyBuilder-ML (SAbPred). Predicted 3D structures of antibody paratopes allowed visualization of the Antigen Contact Structures.

### Epitope Mapping by Dot Blotting

A library of ten synthetic peptides (P1-P10, fig. S1B) corresponding to C-terminus portion of TN protein (fig. S1A) were spotted (0.1 μg in 2.5 μl) onto nitrocellulose membranes (Thermo Scientific, Cat No. 88013). Membranes were blocked with 5% milk in PBS-T (PBS with 0.1% Tween-20) and then incubated with anti-TN monoclonal antibodies (mAb8 or mAb9, 1.0 μg/mL). After washing, membranes were incubated with HRP-conjugated goat anti-mouse IgG (Jackson ImmunoResearch), and signals were detected using chemiluminescence as described ^6,30^.

### Surface Plasmon Resonance (SPR) Assay

Binding kinetics between HMGB1 and P2–1 were measured using Nicoya Life Science gold nanoparticle-based OpenSPR technology (Kitchener, ON, Canada) as previously described ^6,84^. Recombinant human HMGB1 was immobilized onto a Carboxyl sensor chip, and P2–1 peptide was flowed over immobilized HMGB1 at various concentrations in HBS-P buffer (GE Healthcare). Response units were recorded over time, and binding affinity was estimated as the equilibrium dissociation constant *K*_D_ using Trace Drawer Kinetic Data Analysis v.1.6.1 (Nicoya Life Science).

### P2 Peptide Structure Prediction and ClusPro Protein-Protein Docking

To predict the three-dimensional (3D) structure of the P2 peptide, its amino acid sequence was submitted in FASTA format to the Iterative Threading ASSembly Refinement (I-TASSER) web server (https://zhanggroup.org/I-TASSER/). I-TASSER predicted peptide 3D structures by integrating template-based threading, *ab initio* modeling, and molecular dynamics simulations to construct and refine models ^85^. It generates several predicted models, each accompanied by a C-score ranging from −5 to 2, where higher values indicate higher confidence and better model quality. To elucidate P2/HMGB1 interaction details, the binding interface between HMGB1 B-box and P2 was predicted using the ClusPro Web Server (https://cluspro.org). The crystal structure of HMGB1 B-box (PDB: 1HME) was used as the receptor, and the I-TASSER predicted structure of P2 peptide (fig. S2A, Model 1) was used as the ligand. The docking algorithm explored various binding modes, and the complex with the lowest Gibbs free energy was selected for visualization and analysis of potential interaction sites.

### Human Peripheral Blood Mononuclear Cell (PBMC) Isolation and Culture

Human blood was purchased from the New York Blood Center (Long Island City, NY, USA), and PBMCs were isolated by density gradient centrifugation through Ficoll (Ficoll-Paque PLUS) as previously described ^6,30^. PBMCs were cultured in RPMI-1640 supplemented with 10% human serum, 2 mM L-glutamine, 100 U/mL penicillin, and 100 μg/mL streptomycin (all from Invitrogen). For experiments, PBMCs were stimulated with recombinant human HMGB1 (0.5 or 2.0 μg/ml) for 16 hours, either alone or in combination with P2–1 at 5.0 or 10.0 μg/ml. Cell-free supernatants were collected for analysis of cytokines/chemokines levels by Cytokine Antibody Arrays, or pCTS-L levels by Western blotting analysis as described ^6^.

### RNA Sequencing (RNA-Seq) and Bioinformatics Analysis

Total RNA was isolated from human PBMCs stimulated with HMGB1 in the absence or presence of P2–1 (5.0 or 10.0 μg/ml) using the RNeasy Mini Kit (Qiagen) according to the manufacturer’s instructions. RNA quantity and quality were assessed using a NanoDrop spectrophotometer and an Agilent Bioanalyzer. cDNA libraries were prepared using the TruSeq RNA Library Prep Kit v2 (Illumina) and sequenced on an Illumina NovaSeq 6000 platform to generate 100 bp paired-end reads. Raw sequencing reads were quality-checked using FastQC, trimmed with Trimmomatic, and aligned to the human reference genome (GRCh38) using HISAT2. Gene counts were quantified using StringTie and DESeq2 was used for differential gene expression analysis. Volcano plots and heatmaps were generated using ggplot2 and pheatmap packages in R, respectively. Genes with an adjusted *P*-value < 0.05 and a |log2-fold change| > 1 were considered differentially expressed.

### Cytokine Antibody Array Analysis

Levels of cytokines and chemokines in mouse serum and joint tissue lysates, or human PBMC culture supernatants were determined using murine or human Cytokine Antibody Arrays (e.g., Cat. No. M0308003 or Cat. No. AAH-CYT-3–4, RayBiotech Inc., Norcross, GA, USA) as previously described ^6,30,86,87^. Serum samples were collected from mice at 24 h post-CLP. The soft tissue of the hindpaw and ankle joints was carefully collected using a previously described protocol ^88^. Briefly, 0.5 g of fresh soft tissue was cut into 3 mm pieces, and mixed with 400 μL of a hypertonic solution (500 mL PBS with 4.5 g NaCl and 2× protease inhibitor cocktail) in a 10 mL syringe. Mechanical extrusion, involving 30 rapid back-and-forth movements of the plunger, was then performed. The expelled extracellular fluid was collected, immediately aliquoted, and stored at −20°C until analysis. Cell-conditioned medium was collected from PBMC cultures after 16 h of stimulation. Arrays were performed according to the manufacturer’s protocol, followed by chemiluminescent detection and densitometric quantification as previously described ^6,30^.

### Western Blot Analysis for pCTS-L

Cell-conditioned medium from human PBMC cultures was collected, and concentrated using Amicon Ultra Centrifugal Filters. Equal volumes of conditioned medium, representing equivalent numbers of cells, were resolved on sodium dodecyl sulfate (SDS)-polyacrylamide gels and transferred to polyvinylidene difluoride (PVDF) membranes. After blocking with 5% nonfat milk in Tris-buffered saline with 0.1% Tween 20 (TBST), membranes were probed with a home-made primary monoclonal antibody against pCTS-L (e.g., mAb2, 1:1000) overnight as previously described ^6^. After washing, membranes were incubated with a horseradish peroxidase-conjugated secondary antibody. Protein bands were visualized using enhanced chemiluminescence (ECL) substrate (Pierce) and detected with a chemiluminescence imaging system (e.g., Bio-Rad ChemiDoc MP). The relative levels of specific proteins were determined using the UN-SCAN-IT Gel Analysis Software Version 7.1 (Silk Scientific Inc., Orem, UT, USA) and expressed in arbitrary units (AU).

### Statistical Analysis

All data were first assessed for normality using the Shapiro-Wilk test before applying the appropriate statistical analyses. All data are presented as mean ± standard error of the mean (SEM) unless otherwise specified. For comparison among multiple groups with non-normal (skewed) distribution, the Kruskal-Wallis ANOVA test followed by Dunn’s post-hoc test was used to evaluate statistical differences. Survival curves were analyzed using Kaplan-Meier method and compared using the nonparametric log-rank (Mantel-Cox) test. Statistical significance was defined as a *P* value less than 0.05.

## Supplementary Material

Supplement 1

Supplementary Files

This is a list of supplementary files associated with this preprint. Click to download.

• DataFileS2.txt

• DataFileS3.txt

• DataFileS19252025.xlsx

**Data File S1.** Primary data.

**Data File S2.** PDB files for the predicted P2 structure

**Data File S3.** PDB files for the docking model of the HMGB1 B-box/P2 complex.

## Data Availability

All data needed to evaluate the conclusions in the paper are present in the paper and the Supplementary Materials.
