# Supplementary material for: Repurposing a Detrimental Antibody Epitope as Targeted Therapeutics for Sepsis and Rheumatoid Arthritis
Source: Res Sq. 2025 Sep 19:rs.3.rs-7633404. Preprint. [Version 1] doi: 10.21203/rs.3.rs-7633404/v1 (PMC12458545; doi:10.21203/rs.3.rs-7633404/v1)
Supplement: Supplement 1 [file NIHPPRS7633404V1-supplement-1.pdf]

# Supplementary Materials for

# Repurposing a Detrimental Antibody Epitope as Targeted Therapeutics for Sepsis and Rheumatoid Arthritis

Weiqiang Chen [1, 2†], Li Lou [1†], Xiaoling Qiang [1, 2†], Cassie Shu Zhu [1, 2†], Jianhua Li [1], Shujin Chen [1], Brian Xiong [1], Huan Yang [1], Ping Wang [1, 2], Kevin J. Tracey [1, 2], and Haichao Wang [1, 2*]

† equally contributed

* Corresponding author: E-mail: hwang@northwell.edu

# Supplemental Figures

## Figure S1

**A** Amino acid sequence of human and murine tetranectin (TN) protein

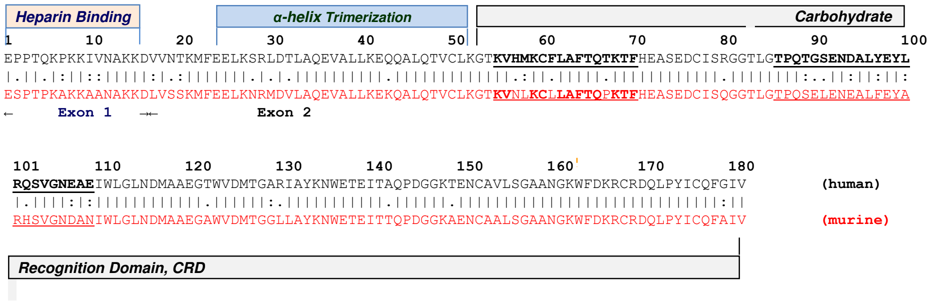


**B** Sequence of synthetic peptides

| Peptide # | Sequence |
|---|---|
| P1 | 46-LQTVCLKGT-54 |
| P2 | 55-KVHMKCFLAFTQTKTF-70 |
| P3 | 71-HEASEDCISRGG-83 |
| P4 | 81-GGTLGTPQTG-90 |
| P5 | 86-TPQTGSENDALYEYLRQSVGNEAE-109 |
| P6 | 105-GNEAEIWLGLNDMAAEGT-122 |
| P7 | 121-GTWVDMTGARIAYKNWETEITAQP-144 |
| P8 | 140-ITAQPDGGKTENC-152 |
| P9 | 151-NCAVLSGAANGKWFDKR-167 |
| P10 | 158-AANGKWFDKRCRDQLPYICQFGIV-181 |

**C** Expression of ΔTN

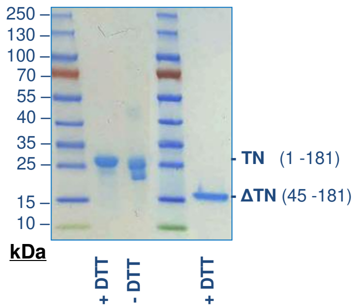


**D** Effect of ΔTN on sepsis

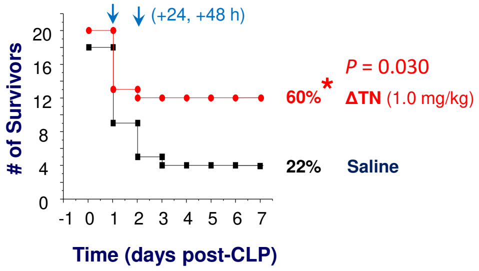

**Figure S1**. **A tetranectin (TN) mutant lacking the N-terminal α-helix trimerization domain retained TN's protective efficacy in sepsis**.

**A**) Domain architecture of human and murine TN, beginning after the N-terminal 21-amino acid leader signal sequence.

**B**) Sequences of ten synthetic peptides derived from the human TN carbohydrate recognition domain (CRD), including the P2 epitope, used for functional screening.

**C**) Expression and purification of recombinant full-length human TN (residues 1-181) and its N-terminal deletion mutant (ΔTN, residues 45-181). Both constructs with a N-histidine tag were expressed in *E. coli* BL21 (DE3) pLysS cells as insoluble inclusion bodies, which were isolated by differential centrifugation following extensive washing in 1 × PBS containing 1% Triton X-100. The inclusion bodies were then solubilized in 8 M urea, and refolded by dialysis in 10 mM Tris buffer (pH 8.0) containing N-lauroylsarcosine. Subsequently, the recombinant proteins were subjected to extensive Triton X-114 extractions to remove contaminating endotoxins.

**D**) Recombinant ΔTN conferred significant protection against lethal sepsis. Male (n = 10) and female (n = 10) Balb/C mice were subjected to cecal ligation and puncture (CLP). Recombinant ΔTN was given intraperitoneally at 24 h and 48 h post-CLP, and animal survival rates were monitored. *, $P < 0.05$ versus saline control group.

## Figure S2

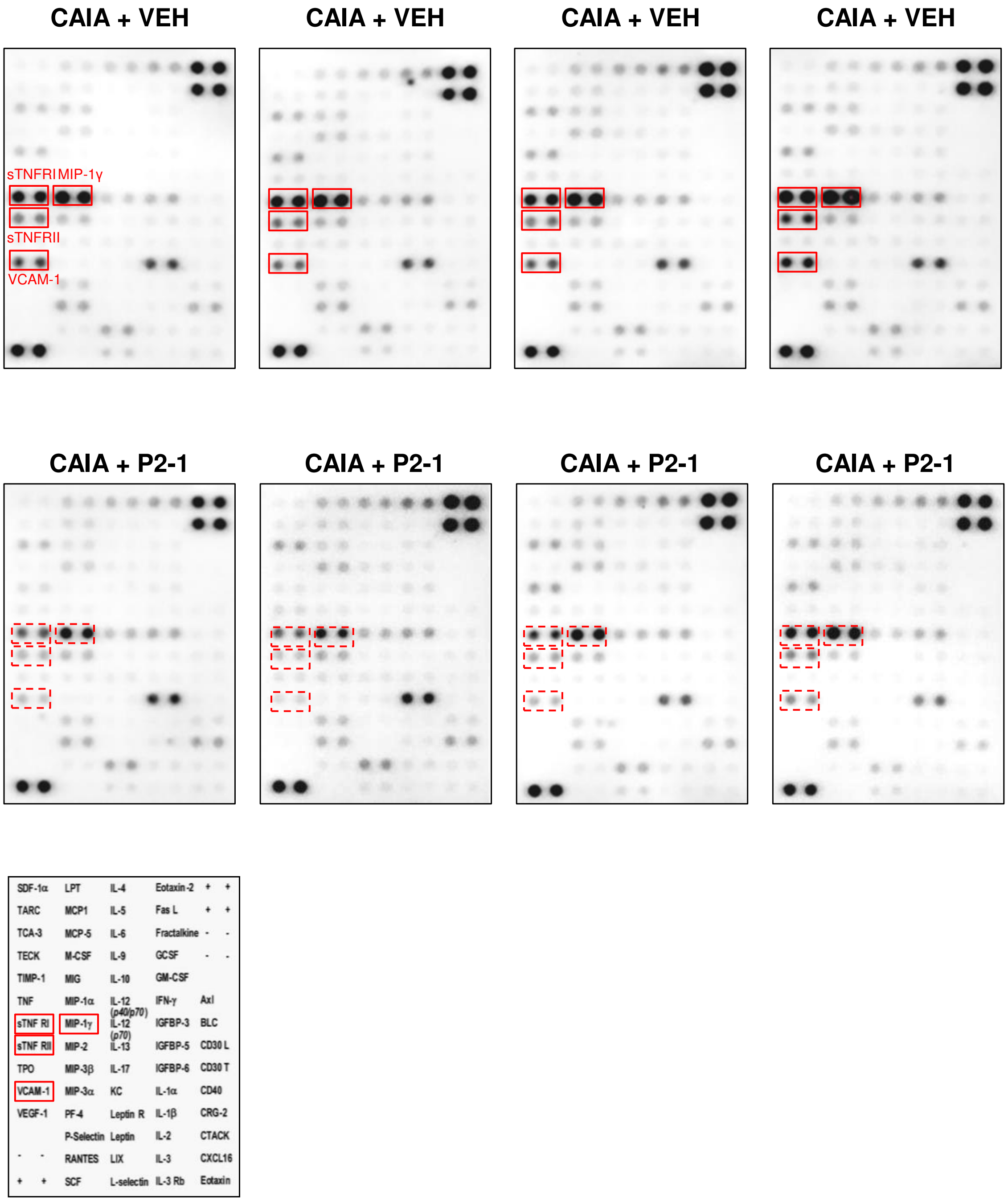


**Figure S2. Representative Cytokine Antibody Arrays depicting the effect of P2-1 on CAIA-induced joint inflammation.**

Representative images from cytokine antibody arrays of joint tissue lysates from four vehicle-treated and four P2-1-treated CAIA mice (harvested Day 10). These demonstrate the visible reduction in various inflammatory cytokines and chemokines in P2-1-treated samples, supporting the quantitative data in Figure 4D.

## Figure S3

**A** **P2 Structure Prediction [Model # (C-Score)]**

**# 1 (- 1.37)** **# 2 (- 3.29)** **#3 (- 3.73)** **# 4 (- 1.85)**

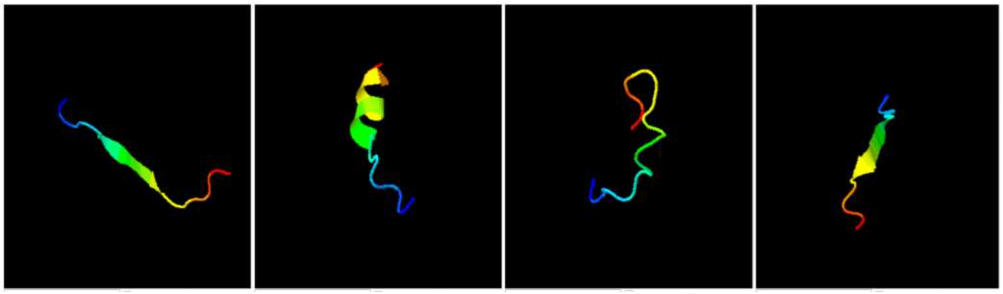

**B** **HMGB1/P2 Docking [Model # (Gibbs Free Energy, KJ/mol)]**

**# 0 (- 663.0)** **# 2 (- 646.8)** **# 4 (- 738.5)** **# 6 (- 685.6)** **# 8 (- 667.4)**

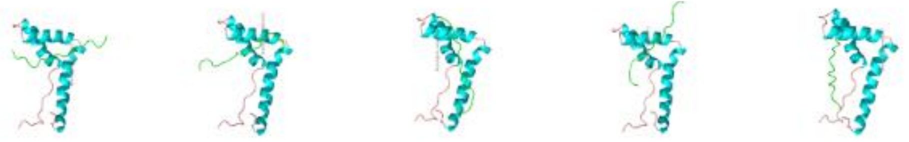

**# 1 (- 713.1)** **#3 (- 691.3)** **# 5 (- 653.0)** **# 7 (- 666.9)** **# 9 (- 649.7)**

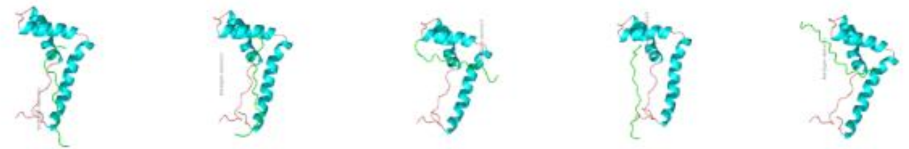

**C** **HMGB1-inderacting domains or residues for P2 Peptide**

```
1           11          21          31          41          51          61          71
MGKGDPKKPRGKMSSYAFFVQTCREEHKKKHPDASVNFSEFSKKCSERWKTMSAKEKGKFEDMAKADKARYEREMKTYIP

                                               "B-box" (PDB: 1HME)
81          91          101         111         121         131         141         151
PKGETKKKFKDPNAPKRPPSAFFLFCSEYRPKIKGEHPGLSIGDVAKKLGEMWNNTAADDKQPYEKKAAKLKEKYEKDIA
                                                  "P2-binding residue:Model#4"
        "TLR4-binding domain"                                                "RAGE-binding

161         171         181         191         201         211
AYRAKGKPDAAKKGVVKAEKSKKKKEEEEDEEDEEDEEEEEDEEDEDEEEDDDDD                  (Human HMGB1)
       Domain"


1           11          21          31          41          51          61          71
FKDPNAPKRPPSAFFLFCSEYRPKIKGEHPGLSIGDVAKKLGEMWNNTAADDKQPYEKKAAKLKEKYEKDIAAYRAK  (B-box)
                                            43 46 "P2-binding"    64  68
                                       KVHM--KCFLA-------------FQTKTF           (P2)
"TLR4-binding domain"                                         "RAGE-binding"
```

**Figure S3. Predicted P2 peptide structure and its Docking interaction with HMGB1 B-box.**

**A)** Predicted 3D structure of the P2 peptide. The model was generated using the I-TASSER (Iterative Threading ASSembly Refinement) web server (https://zhanggroup.org/I-TASSER/). Model confidence is indicated by its C-score, where higher values denote greater confidence and better model quality.

**B)** ClusPro Protein-Protein Docking of HMGB1 B-box and P2**.** The interaction between human HMGB1 B-box (PDB: 1HME) and the P2 peptide was modeled using the ClusPro web server (https://cluspro.bu.edu/login.php). Docking models are ranked by their estimated binding free energy (Gibbs Free Energy), with lower values signifying higher confidence.

**C)** Interfacial residues in the HMGB1/P2 complex. Key interacting residues are highlighted in **red** within HMGB1 and B-box sequences. The P2-binding residues are distant from its TLR4-binding domain (residues 89-108) but partially overlaps with the RAGE-binding domain (residues 150-183, including E153 and K154). This spatial arrangement suggests that P2 or P2-1 may selectively interfere with RAGE-dependent HMGB1 activities (e.g., endocytosis and macrophage pyroptosis induction) without affecting its TLR4-dependent functions (e.g., the induction of cytokines and chemokines)

# Figure S4

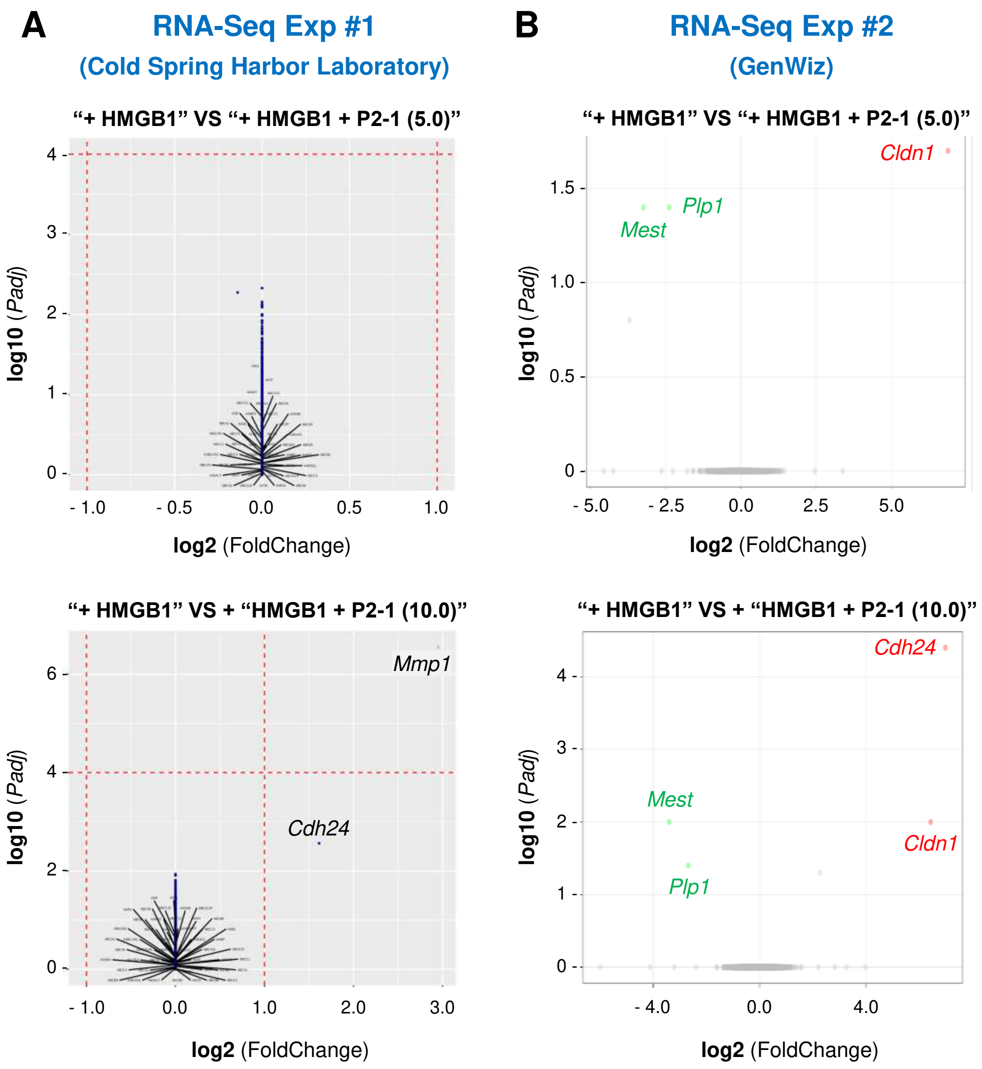


**Figure S4. Volcano plots illustrating P2-1-modulated differential gene expression in HMGB1-stimulated human PBMCs.**

Data were generated from two distinct RNA-seq studies performed at Cold Spring Harbor Laboratory and GenWiz, respectively. Volcano plots were used to identify genes significantly up- or down-regulated by P2-1 in human PBMCs stimulated with HMGB1. The X-axis represents the log2 fold change (log2 FoldChange) in gene expression between PBMCs treated with HMGB1 alone and those treated with HMGB1 + P2-1 at 5.0 µg/ml (**Top Panel**) or 10.0 µg/ml (**Bottom Panel**). The Y-axis represents the statistical significance as the -log10 (adjusted p-value) for the observed expression change of each gene. The observed tight clustering of most data points around a 0 log2FC and near-zero -log10(*P*adj) implies that P2-1 does not induce widespread reversal of HMGB1-orchestrated transcriptional changes.

# Figure S5

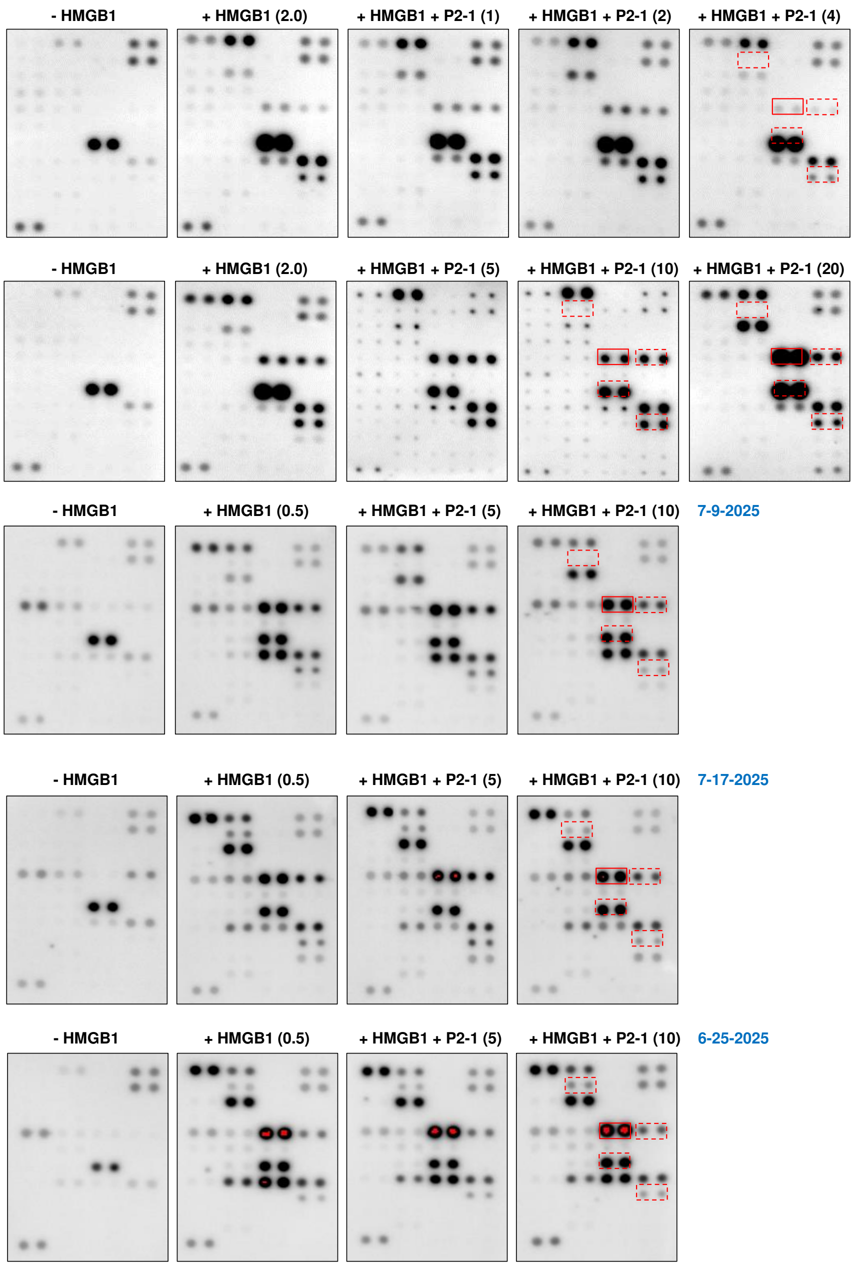


**Figure S5. Representative Cytokine Antibody Array data illustrating the effect of P2-1 on HMGB1-induced cytokines and chemokines.**

Human PBMCs were stimulated with HMGB1 (0.5 or 2.0 µg/ml) in the absence or presence of P2-1 (5.0 and 10.0 µg/ml) for 16 h, the extracellular levels of cytokines and chemokines were determined by Cytokine Antibody Arrays. Comprehensive cytokine antibody array results showing that P2-1 (5.0 and 10.0 µg/ml) does not broadly inhibit the HMGB1-induced (0.5 or 2.0 µg/ml, 16h) secretion of most inflammatory cytokines and chemokines from human PBMCs.

## Figure S6

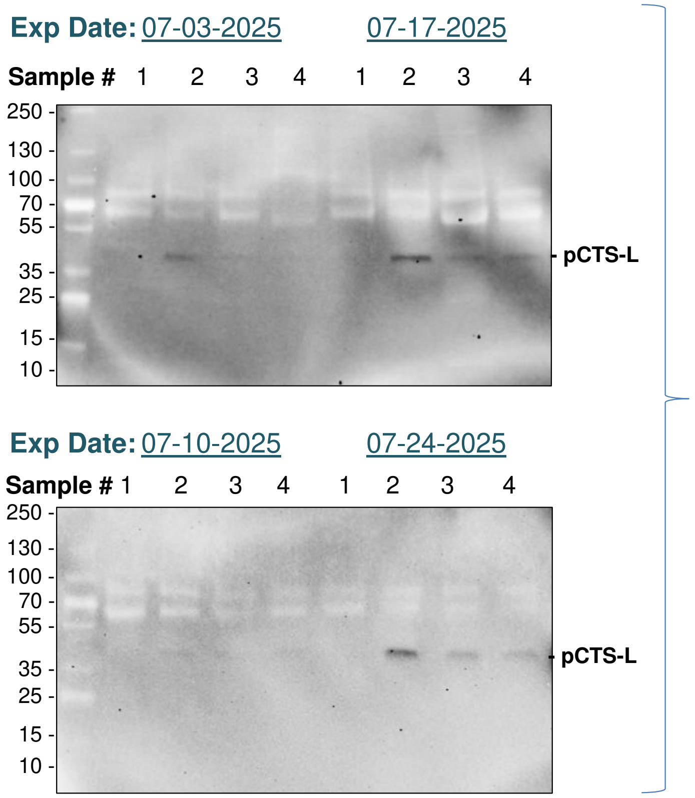


1 - HMGB1

2 + HMGB1 (0.5 μg/ml)

3 + HMGB1 (0.5 μg/ml) + P2-1 (5.0 μg/ml)

4 + HMGB1 (0.5 μg/ml) + P2-1 (10.0 μg/ml)

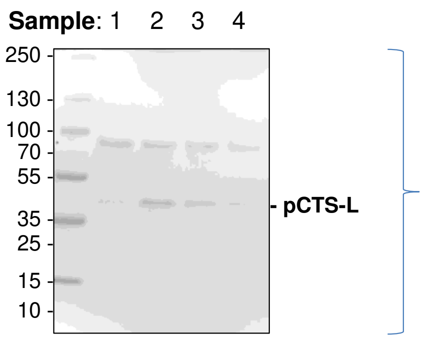


1 - HMGB1

2 + HMGB1 (2.0 μg/ml)

3 + HMGB1 (2.0 μg/ml) + P2-1 (5.0 μg/ml)

4 + HMGB1 (2.0 μg/ml) + P2-1 (10.0 μg/ml)

**Figure S6. Full Western blot analysis of P2-1 effect on HMGB1-induced pCTS-L release in human PBMCs.**

Human PBMCs were stimulated with HMGB1 (0.5 or 2.0 μg/ml) in the absence or presence of P2-1 (5, 10, and 20 μg/ml) for 16 h, the levels of pCTS-L in the cell-conditioned medium were determined by Western blotting, with sample loading normalized by volume of culture medium conditioned by an equivalent number of cells. Representative Western blot images showing the dose-dependent inhibitory effect of P2-1 (5.0 and 10.0 μg/ml) on HMGB1-induced (0.5 or 2.0 μg/ml) pCTS-L secretion from human PBMCs.

# Figure S7

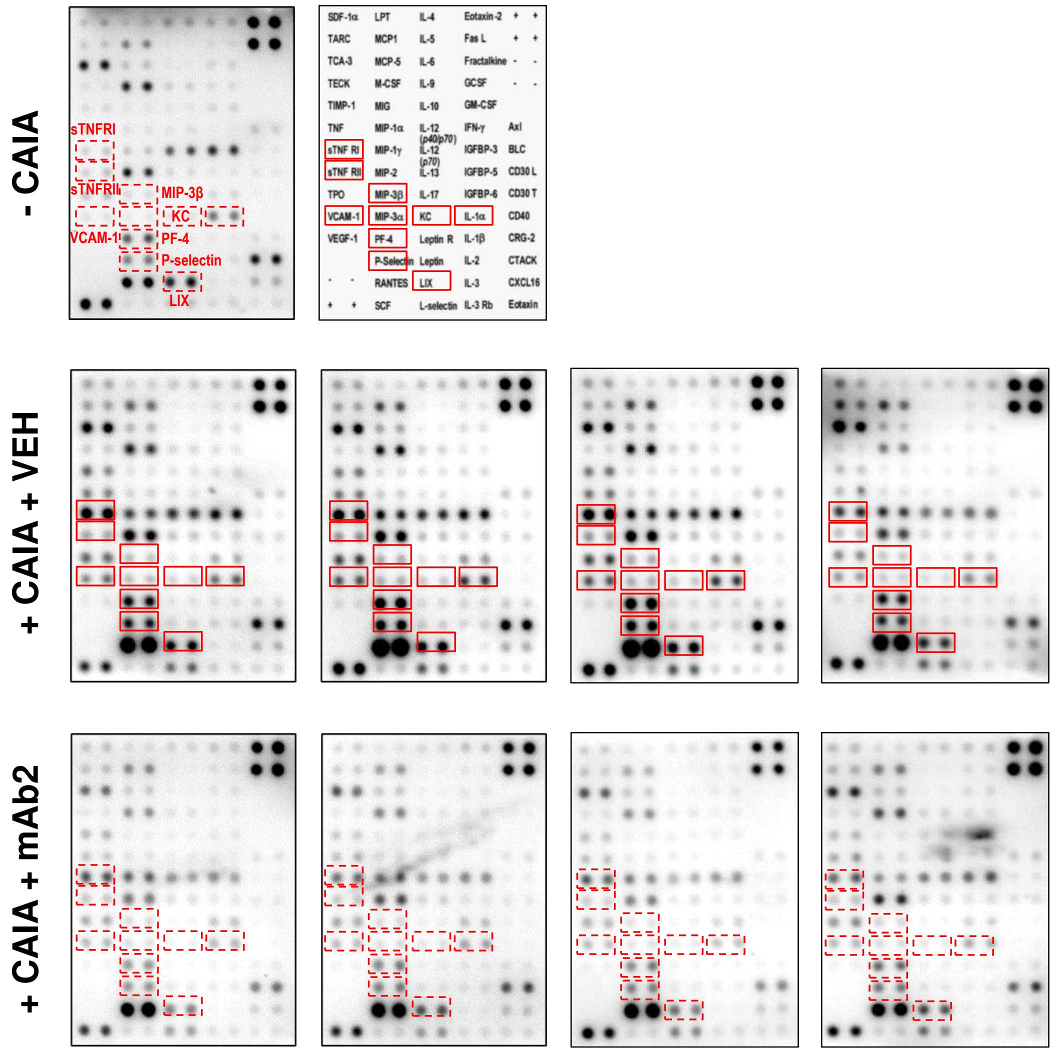


**Figure S7. Representative Cytokine Antibody Arrays illustrating the effect of mAb2 on CAIA-induced joint inflammation.**

Joint soft tissue from Balb/C mice, subjected to CAIA and treated with a pCTS-L-neutralizing mAb2 (intraperitoneally on Day 6, 7, 8, 9), was harvested on Day 10. Levels of various cytokines and chemokines were measured using Cytokine Antibody Arrays to assess inflammation. Representative images from cytokine antibody arrays of joint tissue lysates from a normal mouse ("- CAIA"), four vehicle (VEH)-treated CAIA mice, and four pCTS-L-neutralizing mAb2-treated CAIA mice (harvested Day 10). These show the visual reduction in various inflammatory cytokines and chemokines in mAb-treated samples.

## Figure S8

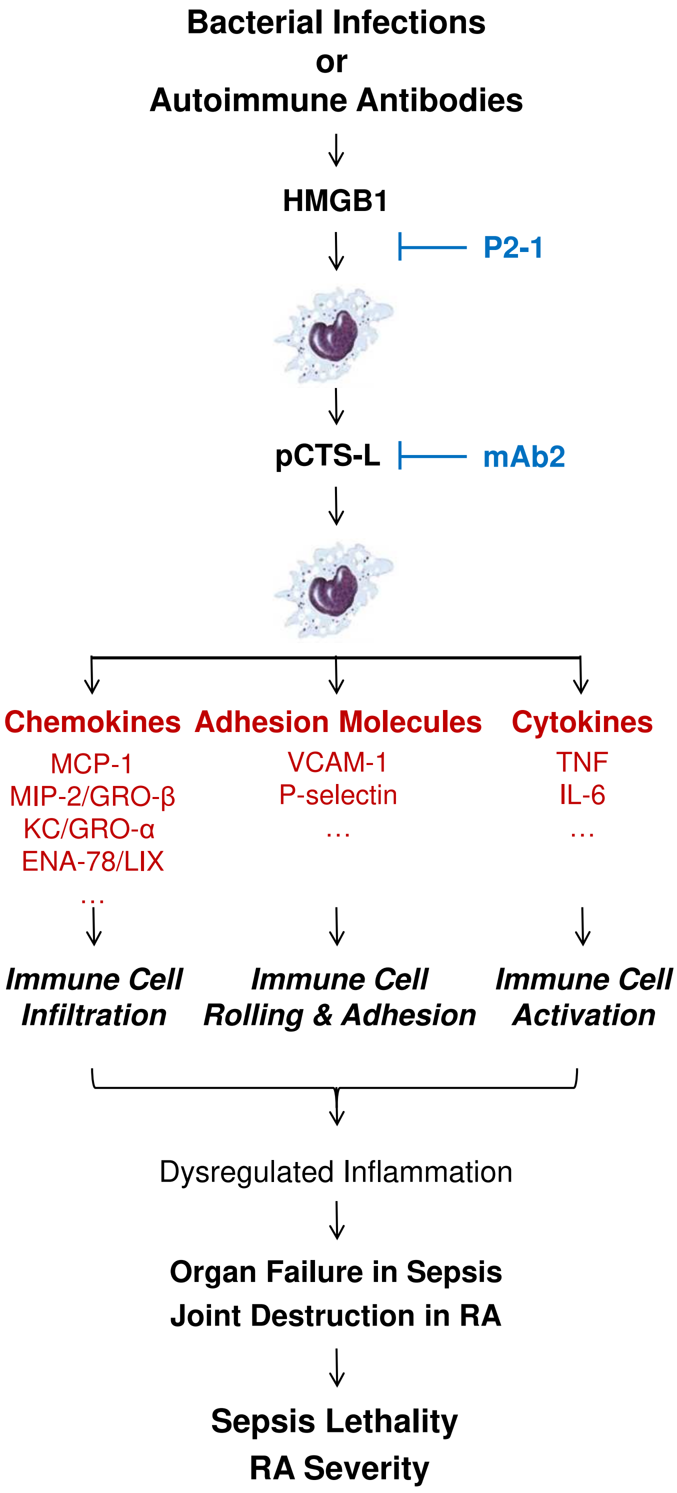
Bacterial Infections
or
Autoimmune Antibodies
HMGB1
P2-1
pCTS-L
mAb2
Chemokines
MCP-1
MIP-2/GRO-β
KC/GRO-α
ENA-78/LIX
…
Adhesion Molecules
VCAM-1
P-selectin
…
Cytokines
TNF
IL-6
…
Immune Cell Infiltration
Immune Cell Rolling & Adhesion
Immune Cell Activation
Dysregulated Inflammation
Organ Failure in Sepsis
Joint Destruction in RA
Sepsis Lethality
RA Severity

**Figure S8. Proposed model for P2-1-mediated intervention in the inflammatory HMGB1-pCTS-L axis in sepsis and rheumatoid arthritis.**

Pathogenic inflammation in sepsis and rheumatoid arthritis (RA) is characterized by a dysregulated innate immune response, involving complex interactions between chemokines, adhesion molecules, and pro-inflammatory cytokines. Chemokines (e.g., CCL20, CXCL1) guide immune cell infiltration to inflamed sites, while adhesion molecules (e.g., VCAM-1, P-selectin) facilitate their extravasation. Key cytokines, including TNF, HMGB1, and pCTS-L, act through specific receptors (e.g., TNFRI/II, TLR4, RAGE) to perpetuate a self-amplifying inflammatory cycle, ultimately causing organ failure in sepsis or joint destruction in RA. This figure illustrates how P2-1, a peptide derived from a detrimental anti-TN mAb9, directly binds HMGB1 and selectively inhibits HMGB1-induced *Ctsl* mRNA upregulation and pCTS-L secretion. Given the critical role of pCTS-L in lethal sepsis (Chen et al., ***Science Advances***, 2023) and its confirmed contribution of arthritis pathogenesis (as demonstrated herein), P2-1 emerges as a targeted therapy specifically disrupting the HMGB1-pCTS-L axis in inflammatory diseases.

# Supplemental tables

## Tabel S1. Key Reagent Source Table

| REAGENT or RESOURCE | SOURCE | IDENTIFIER |
|---|---|---|
| **Antibodies** | | |
| Mouse anti-β-actin antibody | Sigma-Aldrich | Cat. # A1978 |
| Mouse anti-murine procathepsin L (pCTS-L) monoclonal antibodies | Home-made | *Science Advances*, 2023 |
| Mouse anti-human tetranectin mAb8 and mAb9 | Home-made | *Science Translational Medicine*, 2000 |
| HRP-conjugated mouse IgG kappa-binding protein (m-IgGκ BP) | Santa Cruz | Cat. # sc-516102 |
| Anti-Type II Collagen Antibody Cocktail (A2-10, D1-2G, D8-6, F10-21) | Chondrex Inc. | Cat. #53100 |
| **Chemicals** | | |
| Crude bacterial endotoxin (lipopolysaccharide, LPS) | Sigma-Aldrich | *E. coli 0111:B4* |
| Human serum | Sigma-Aldrich | Cat. # H3667 |
| Recombinant human HMGB1 protein | Home-made | *Science*, 1999 |
| Dulbecco's modified Eagle medium (DMEM) | Invitrogen/Life Technologies | Cat. # 11995-065 |
| OPTI-MEM I Reduced-Serum Medium | Thermo Fisher Scientific | Cat. # 31985062 |
| Penicillin/streptomycin | Invitrogen/Life Technologies | Cat. # 15140-122 |
| Ketamine | Henry Schein Special Market | Cat. # 2480861 |
| Xylazine | Sigma-Aldrich | Cat. # X1251 |
| **Critical commercial assays and SPR Sensor Chip** | | |
| Murine Cytokine Antibody Arrays | RayBiotech Inc | Cat. #. M0308003 |
| Human Cytokine Antibody C3 Arrays | RayBiotech Inc | Cat. # AAH-CYT-3-4 |
| Nylon Von Frey filaments | Stoelting Co. | Cat. #58011 |
| High Sensitivity Carboxyl Sensors | Nicoya Lifesciences | Cat. # SEN-HS-8-COOH |
| **Experimental models: Organisms/strains** | | |
| Balb/C mice | Jackson Laboratory | Stock # 000651 |
| Balb/C mice | Charles River Laboratories | Strain Code: 194 |
| **Software and algorithms** | | |
| UN-SCAN-IT Gel Analysis Software Version 7.1 | Silk Scientific Inc. | |
